# Preparation, Identification, and Activity Evaluation of Eight Antioxidant Peptides from Protein Hydrolysate of Hairtail (*Trichiurus japonicas*) Muscle

**DOI:** 10.3390/md17010023

**Published:** 2019-01-02

**Authors:** Xiu-Rong Yang, Lun Zhang, Dong-Ge Ding, Chang-Feng Chi, Bin Wang, Jian-Cong Huo

**Affiliations:** 1 Zhejiang Provincial Engineering Technology Research Center of Marine Biomedical Products, School of Food and Pharmacy, Zhejiang Ocean University, 1st Haidanan Road, Zhoushan 316022, China; yxr1948008999@163.com (X.-R.Y.); Zl15525864652@163.com (L.Z.); 9001000@163.com (D.-G.D.); yujia0112@sina.com (J.-C.H.); 2 National and Provincial Joint Laboratory of Exploration and Utilization of Marine Aquatic Genetic Resources, National Engineering Research Center of Marine Facilities Aquaculture, School of Marine Science and Technology, Zhejiang Ocean University, 1st Haidanan Road, Zhoushan 316022, China

**Keywords:** hairtail (*Trichiurus japonicas*), muscle, peptide, antioxidant activity

## Abstract

In this report, protein of hairtail (*Trichiurus japonicas*) muscle was separately hydrolyzed using five kinds of proteases (alcalase, trypsin, neutrase, pepsin, and papain), and the papain- and alcalase-hydrolysates showed higher 2,2-diphenyl-1-picrylhydrazyl radicals (DPPH•) and hydroxyl radical (HO•) scavenging activity than other three protease hydrolysates. Therefore, the protein hydrolysate of hairtail muscle (HTP) was prepared using binary-enzymes hydrolysis process (papain + alcalase). Subsequently, eight antioxidant peptides were purified from HTP using membrane ultrafiltration and chromatography technology, and their amino acid sequences were identified as Gln-Asn-Asp-Glu-Arg (TJP1), Lys-Ser (TJP2), Lys-Ala (TJP3), Ala-Lys-Gly (TJP4), Thr-Lys-Ala (TJP5), Val-Lys (TJP6), Met-Lys (TJP7), and Ile-Tyr-Gly (TJP8) with molecular weights of 660.3, 233.0, 217.1, 274.1, 318.0, 245.1, 277.0, and 351.0 Da, respectively. TJP3, TJP4, and TJP8 exhibited strong scavenging activities on DPPH• (EC_50_ 0.902, 0.626, and 0.663 mg/mL, respectively), HO• (EC_50_ 1.740, 2.378, and 2.498 mg/mL, respectively), superoxide anion radical (EC_50_ 2.082, 2.538, and 1.355 mg/mL, respectively), and 2,2′-azino-bis-3-ethylbenzothiazoline-6-sulfonic acid (ABTS) radical (EC_50_ 1.652, 0.831, and 0.586 mg/mL, respectively). Moreover, TJP3, TJP4, and TJP8 showed higher reducing power and inhibiting ability on lipid peroxidation in a linoleic acid model system. These results suggested that eight isolated peptides (TJP1 to TJP8), especially TJP3, TJP4, and TJP8 might serve as potential antioxidants applied in the pharmaceutical and health food industries.

## 1. Introduction

Reactive oxygen species (ROS) such as superoxide anion radical ($O_{2}^{-}$•), hydrogen peroxide (H_2_O_2_), hydroxyl radical (HO•), and singlet oxygen (^1^O_2_), are formed in aerobic organisms as a natural by-product of oxygen metabolism and play vital roles in the physiological processes involved in signal transduction and homeostasis [1,2]. Under normal conditions, superfluous ROS are effectively eliminated by antioxidant enzymes and non-enzymatic factors in organisms [3]. An imbalance in pro-oxidant/antioxidant can induce oxidative stress, trigger accumulated ROS production, and result in cell damage and many health disorders, such as diabetes mellitus, coronary heart diseases, cancer, hepatic diseases, and inflammatory diseases [4,5]. Additionally, oxidation is believed to the major course of food deterioration because ROS-mediated oxidation can react with lipids, proteins, amino acids, vitamins, and cholesterol to produce undesirable off-flavors, and potentially toxicity during food processing, transportation, and storage [3,6]. Therefore, it is very important for pharmaceutical, health food, and food processing and preservation industries to develop efficient antioxidants [7]. At present, some artificial antioxidants including butylated hydroxytoluene (BHT), butylated hydroxyanisole (BHA), and tertiary butylhydroquinone (TBHQ) show stronger antioxidant activities and have been widely used in medicine and food industry for retarding oxidation in organisms and food [4,7]. However, the side effects of synthetic antioxidants such as liver damage and carcinogenesis causes consumer anxiety and significantly affect their application [7,8]. Therefore, there has been a major interest in searching for efficient antioxidants from natural sources as alternatives to synthetic antioxidants for countering these adverse effects.

At present, dietary antioxidant ingredients including vitamins, carotenoids, flavonoids, phenols, saccharides, and peptides, have been continually investigated for their health benefits in terms of their scavenging potential of free radicals and low toxicity [9]. Among them, bioactive peptides released from food proteins under controlled proteolysis have aroused wide public concern not only in their possibilities as natural alternatives to synthetic antioxidants, but also for their beneficial effects, lack of residual side effects, and functionality in food systems [10]. 

Antioxidant peptides from food resources are inactive in the amino acid sequence of their parent proteins and are produced by in vitro enzymatic hydrolysis [7]. These peptides with 2 to 20 amino acid residues are considered to be easy absorption and no hazardous immunoreactions. More importantly, antioxidant peptides can exert their activity as free radical scavengers, peroxide decomposers, metal inactivators and oxygen inhibitors to protect food and organisms from ROS [11,12,13]. In recent years, antioxidant peptides derived from seafood were more attractive and have been isolated and identified from diverse marine organisms, such as swim bladders of miiuy croaker (*Miichthys miiuy*) [6], bluefin leatherjacket (*Navodon septentrionalis*) heads and skin [8,14], thornback ray skins [15], *Palmaria palmate* [16], viscera and carcass of Nile tilapia [17], *Pinctada fucata* [18], pectoral fin of salmon [19], and jellyfish gonad [20]. Zhao et al. isolated ten antioxidant peptides from swim bladders of miiuy croaker, and PYLRH and GIEWA exhibited stronger scavenging activities on 2,2-diphenyl-1-picrylhydrazyl radicals (DPPH•), hydroxyl radical (HO•), and superoxide anion radical ($O_{2}^{-}$•) than other eight peptides. Furthermore, FPYLRH and GIEWA could effectively inhibit lipid peroxidation in the β-carotene linoleic acid and in the linoleic acid emulsion system [6]. Harnedy et al. prepared and identified 17 peptides from the macroalgal species *Palmaria palmata*, and SDITRPGGNM showed the highest oxygen radical absorbance capacity and ferric reducing antioxidant power activity with values of 152.43 ± 2.73 and 21.23 ± 0.90 nmol TE/μmol peptide, respectively [16]. MCLDSCLL (P1) and HPLDSLCL (P2) showed potent antioxidant activities against DPPH• and 2,2′-azinobis-(3-ethylbenzothiazoline-6-sulfonic acid) (ABTS) radical (ABTS^+^•) and inhibiting copper-catalyzed human low-density lipoprotein (LDL) oxidation [21]. GAERP, GEREANVM, and AEVG from cartilage protein hydrolysate of spotless smoothhound exhibited good scavenging activities on DPPH•, HO•, ABTS^+^•, and $O_{2}^{-}$•. Furthermore, GAERP, GEREANVM, and AEVG could protect H_2_O_2_-induced HepG2 cells from oxidative stress by decreasing the content of malonaldehyde (MDA) and increasing the levels of superoxide dismutase (SOD), catalase (CAT), glutathione peroxidase (GSH-Px), and glutathione reductase (GSH-Rx) [12]. These studies indicated that seafood-derived peptides had strong antioxidant activity and could be served as ingredients in functional food and food systems to protect food quality by reducing oxidative stress. 

Hairtail (*Trichiurus japonicas*) belongs to cutlassfish family of Trichiuridae and is found throughout tropical and temperate waters worldwide. In China, hairtail is one of the four major aquatic products and wildly distributed in the Yellow Sea, the Bo Hai Sea, and the East China Sea. In our previous research, hairtail hydrolysates chelation with iron (Fe-FPH chelate) had higher hemoglobin regeneration efficiency (HRE), longer exhaustive swimming time, and higher SOD activity. Additionally, Fe-FPH chelate was found to significantly decrease levels the MDA content, visibly enhance the GSH-Px activity in liver and reduce blood lactic acid of rats [22,23]. Therefore, the aim of this work was to (i) optimize the two-step sequential enzymolysis technology; (ii) isolate and identify the antioxidant peptides; and (iii) evaluate the activities of isolated peptides form protein hydrolysate of hairtail muscle in vitro.

## 2. Results and Discussion

### 2.1. Preparation of Protein Hydrolysate from Hairtail (T. japonicas) Muscle (HTP)

#### 2.1.1. Effect of Different Proteases on Protein Hydrolysates from Hairtail (*T. japonicas*) Muscle (HTP)

HO• is a highly reactive radical and can destroy all types of macromolecules such as nucleic acids (mutations), carbohydrates, lipids (lipid peroxidation), proteins and amino acids [24]. DPPH is the traditional and perhaps the most popular standard of the position (g-marker) and intensity of electron paramagnetic resonance (EPR) signals [25]. Therefore, DPPH• and HO• has been widely applied to evaluate the antioxidant ability of compounds to act as free radical scavengers or hydrogen donors [7,25,26].

Chemical treatment, enzymatic hydrolysis, and microbial fermentation of food proteins can be used for bioactive peptides production. However, the enzymatic hydrolysis method is preferred in the food and pharmaceutical industries because the other methods may leave residual organic solvents or toxic chemicals in the final products [7]. Treatment of protein substrate with different proteases will produce several types of protein hydrolysates, which exhibit various extents of antioxidant activities against various antioxidant systems [7,14]. Therefore, the specificity of the enzyme used for the proteolysis is one of the most important factors for the production of bioactive peptides. 

**Figure 1:**
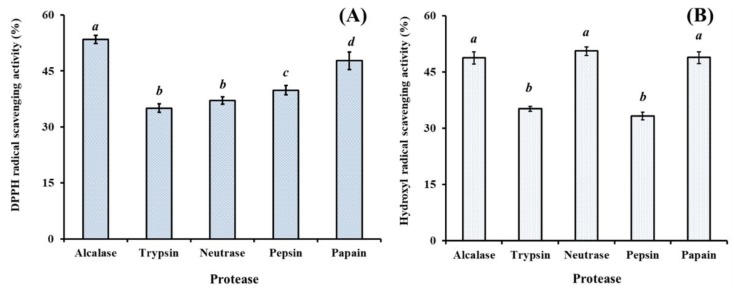
DPPH• (**A**) and HO• (**B**) scavenging activities of different enzymatic hydrolysates from hairtail (*T. japonicas*) muscle at the concentration of 6.0 mg protein/mL. All data are presented as the mean ± SD of triplicate results. *^a^*^–^*^d^* Values with same letters indicate no significant difference of different sample at same concentrations (*p* > 0.05).

In the experiment, defatted proteins of hairtail muscle were separately hydrolyzed with alcalase, trypsin, neutrase, pepsin, and papain at designed conditions and the antioxidant capacities of the resulted hydrolysates at the concentration of 6.0 mg protein/mL were shown in Figure 1. The data indicated that HO• scavenging capacities of the protein hydrolysates were significantly influenced by the type of protease (*p* < 0.05). The DPPH• scavenging activities of alcalase and papain hydrolysates were 53.45 ± 1.05% and 47.75 ± 2.34%, respectively, which were significantly higher than those of trypsin (37.05 ± 0.97%), neutrase (35.05 ± 0.97%), and pepsin (39.85 ± 1.28%) hydrolysates (*p* < 0.05) (Figure 1A). The HO• scavenging activities of neutrase, alcalase, and papain hydrolysates were 50.61 ± 1.16%, 48.76 ± 1.64%, and 48.89 ± 1.58%, respectively, which were significantly higher than those of trypsin (35.21 ± 0.67%) and pepsin (33.29 ± 1.01%) hydrolysates (*p* < 0.05) (Figure 1B). However, there were no significant differences on the HO• scavenging activities of neutrase, alcalase, and papain hydrolysates at the concentration of 6.0 mg protein/mL (*p* > 0.05). In addition, the active sites of papain were lie in basic amino acids, particularly Arg- and Lys-; and the active sites of alcalase were lie in Ala-, Leu-, Val-, Tyr-, Phe-, and Try-. The active sites are significantly different between alcalase and papain, which will help to shorten the hydrolysis time and improve the hydrolysis degree (DH) of protein hydrolysates [7,27,28]. 

**Figure 2:**
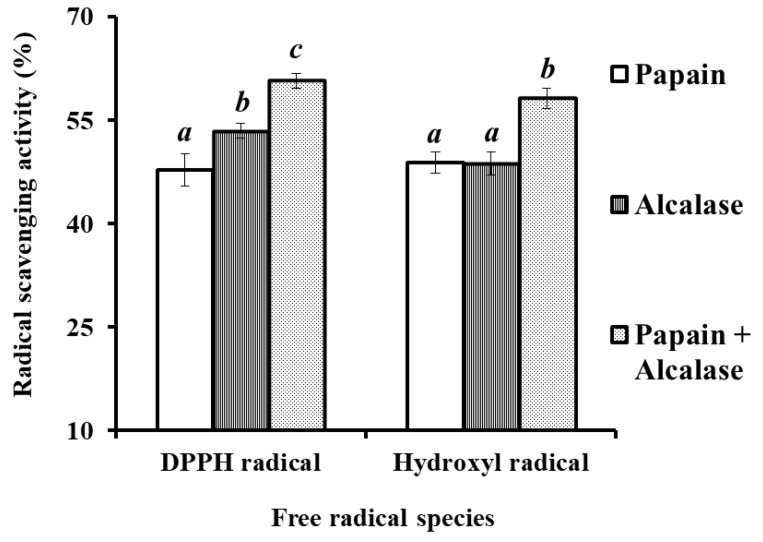
Effect of Papain, Alcalase, and Papain + Alcalase on DPPH• and HO• scavenging activities of protein hydrolysates from hairtail (*T. japonicas*) muscle at the concentration of 6.0 mg protein/mL. All data are presented as the mean ± SD of triplicate results. *^a^*^–^*^c^* Values with same letters indicate no significant difference of different sample at same concentrations *(p* > 0.05).

Under the designed conditions, the protein hydrolysate of hairtail (*T. japonicas*) muscle was prepared using binary-enzymes hydrolysis process (papain + alcalase) and referred to as HTP, and the antioxidant activity of protein hydrolysates was presented in Figure 2. At the concentration of 6.0 mg protein/mL, DPPH• and HO• scavenging activities of HTP were 60.72 ± 1.05% and 58.17 ± 1.53%, respectively, which was significantly higher than those of the hydrolysates prepared separately using papain and alcalase (*p* < 0.05). Therefore, papain and alcalase were selected for the preparation of protein hydrolysate of hairtail muscle.

### 2.2. Purification of Antioxidant Peptides from Protein Hydrolysate from Hairtail (T. japonicas) Muscle (HTP)

#### 2.2.1. Fractionation of HTP Using Membrane Ultrafiltration

**Figure 3:**
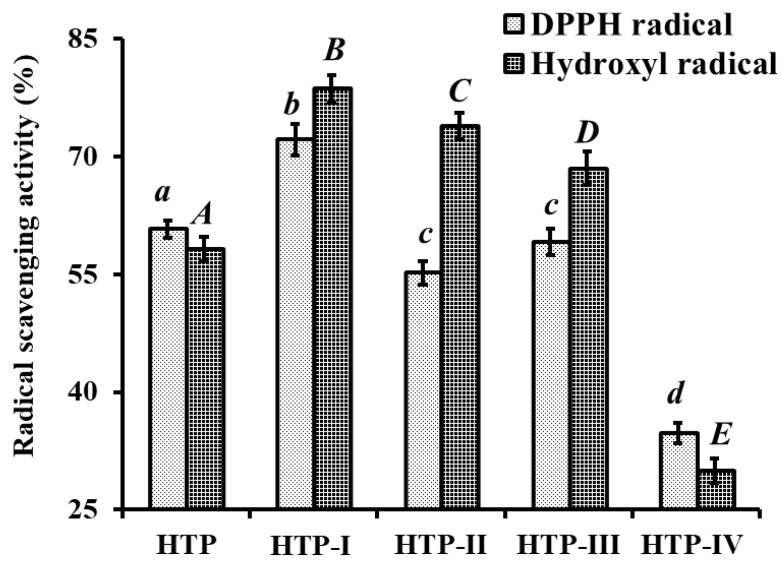
DPPH• and HO• scavenging activity of HTP and its fractions by membrane ultrafiltration at the concentration of 6.0 mg protein/mL. All data are presented as the mean ± SD of triplicate results. *^a^*^–*c*^ or *^A^*^–^*^E^* Column wise values with same superscripts of this type indicate no significant difference (*p* > 0.05).

Membrane ultrafiltration is usually used to enrich the particle sizes of functional molecules and is widely applied in food and beverage processing, biotechnological applications, and pharmaceutical industry [6,7]. Consequently, HTP was divided into four fractions including HTP-І (<1 kDa), HTP-II (1–3 kDa), HTP-III (3–5 kDa), and HTP-IV (>5 kDa) by ultrafiltration with a molecular weight (MW) Cut Off (MWCO) membrane of 1, 3, and 5 kDa. At the concentration of 6.0 mg protein/mL, DPPH• and HO• scavenging activities of HTP-I were 62.13 ± 1.97% and 78.6 ± 1.74%, respectively, which were significantly stronger than those of HTP, HTP-II, HTP-III, and HTP-IV (*p* < 0.05) (Figure 3). The activities of protein hydrolysates and their fractions were affected by the multiple peptides with different chain length and amino acid composition. Sila et al. [7] and Chi et al. [29] reported that MWs of hydrolysates play an important factor in their bioactivities, and hydrolysate fractions with smaller MW showed stronger antioxidant activity than those of larger MW hydrolysates. In the report, HTP-I with short chain peptides showed stronger radical scavenging activity, and this finding was in line with previous reports that the antioxidant abilities of protein hydrolysates were negatively correlated with their average MW [29,30]. Therefore, HTP-I was selected for the subsequent chromatographic separation.

#### 2.2.2. Anion-Exchange Chromatography of HTP-I

Peptides contain acidic and/or hydrophobic amino acid residues such as glutamic acid (Glu), tyrosine (Tyr), methionine (Met), and leucine (Leu), and can be sticks to the anion-exchange resins [31,32]. In addition, acidic and/or hydrophobic amino acid residues in amino acid sequences of peptides will enhance their bioactivities [7,33]. Therefore, anion exchange resins including DEAE-52 cellulose and Q Sepharose FF are usually applied to purified bioactive peptides from protein hydrolysates [8,34,35].

**Figure 4:**
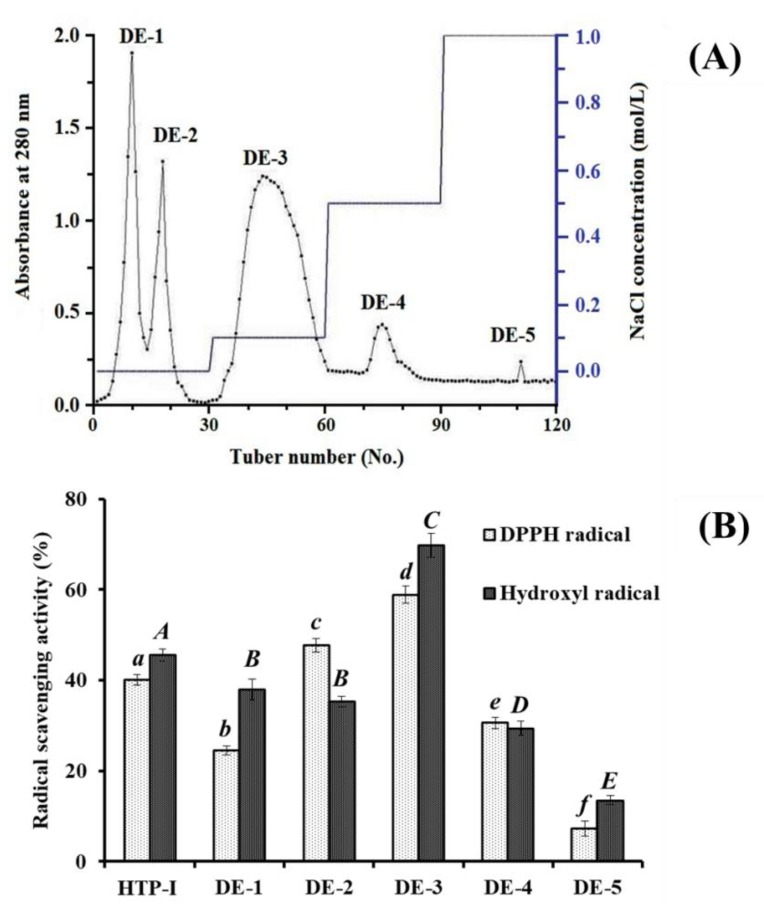
Elution profile of HTP-I in DEAE-52 cellulose anion-exchange chromatography (**A**) and radical scavenging activity of HTP-I and its four subfractions at the concentration of 5.0 mg protein/mL (**B**). All data are presented as the mean ± SD of triplicate results. *^a^*^–^*^f^* or *^A^*^–^*^E^* Values with same superscripts of this type indicate no significant difference (*p* > 0.05).

As shown in Figure 4A, five fractions (DE-1 to DE-5) were separated from HTP-I using a DEAE-52 cellulose column. Amongst them, DE-1 and DE-2 were eluted using deionized water, DE-3 was eluted using 0.1 M NaCl, DE-4 was eluted using 0.5 M NaCl, and DE-5 was eluted using 1.0 M NaCl. DPPH• and HO• scavenging activities of HTP-I and five eluted fractions were showed in Figure 4B, and the results indicated that DPPH• (58.91 ± 1.89%) and HO• (69.74 ± 2.61%) scavenging abilities of DE-3 were significantly stronger than those of HTP-I (DPPH•: 40.13 ± 1.16%; HO•: 45.6 ± 1.35%), DE-1 (DPPH•: 24.51 ± 1.06%; HO•: 37.9 ± 2.33%), DE-2 (DPPH•: 47.7 ± 1.51%; HO•: 35.29 ± 1.18%), DE-4 (DPPH•: 30.64 ± 1.25%; HO•: 29.39 ± 1.63%), and DE-5 (DPPH•: 7.29 ± 1.67%; HO•: 13.51 ± 0.99%) at the concentration of 5.0 mg protein/mL (*p* < 0.05). The results indicated that the highest antioxidant activity of the peptides obtained in DE-3 might be due to the acidic amino acid residues in their peptide sequences. Therefore, DE-3 was selected for the following experiment.

#### 2.2.3. Gel Filtration Chromatography (GFC) of DE-3

GFC is a well-accepted separated technique on the basis of molecular size and usually applied to either fractionate molecules and complexes in a sample into fractions with a particular size range, or remove salt from a preparation of macromolecules [3,36]. Therefore, GFC is often used to separate peptides from protein hydrolysates and their fractions [6,7].

**Figure 5:**
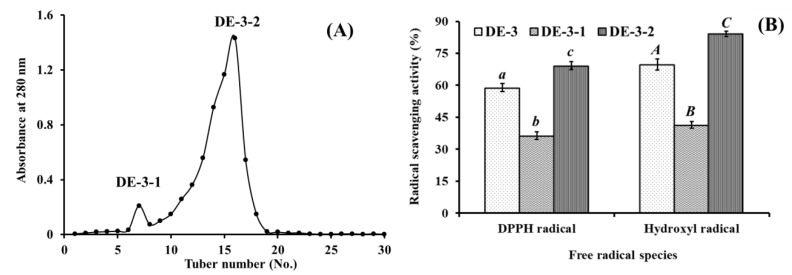
Elution profile of DE-3 in Sephadex G-15 chromatography (**A**) and radical scavenging activities of DE-3 and its fractions at 5.0 mg protein/mL concentration (**B**). All data are presented as the mean ± SD of triplicate results. *^a^*^–*c*^ or *^A^*^–^*^C^* Column wise values with same superscripts of this type indicate no significant difference (*p* > 0.05).

As shown in Figure 5A, DE-3 was separated into two fractions of DE-3-1 and DE-3-2 using a Sephadex G-15 column, and each fraction was collected, lyophilized, and then evaluated for DPPH• and HO• scavenging activity. Figure 5B indicated that DPPH• and HO• scavenging activities of DE-3-2 were 69.21 ± 0.91% and 84.16 ± 1.26% at the concentration of 5.0 mg protein/mL, which were significantly higher than those of DE-3 (DPPH• 58.91 ± 1.89%; HO• 69.74 ± 2.61%) and DE-3-1 (DPPH• 36.27 ± 0.79%; HO• 41.38 ± 1.49%) (*p* < 0.05). Therefore, fraction DE-3-2 was selected for the following isolation process.

#### 2.2.4. Isolation of Peptides from DE-3-2 by Reverse-Phase High Performance Liquid Chromatography (RP-HPLC)

**Figure 6:**
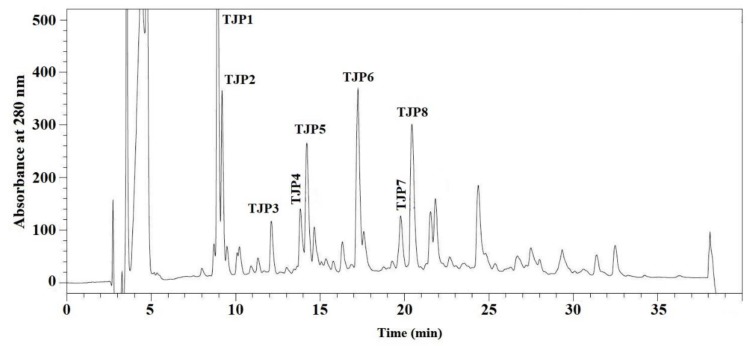
Elution profile of DE-3-2 separated by RP-HPLC system on a Zorbax, SB C-18 column (4.6 × 250 mm) from 0 to 40 min.

RP-HPLC is an effective technique applied to purify and quantify peptides in a mixture solution on their hydrophobic character [37]. The retention time (RT) can qualitatively analyze the isolated peptide and adjusted by changing the ratio of methanol or acetonitrile in mobile phase, and the peak area can be used for quantitative analysis of the isolated peptide [3,38]. As shown in Figure 6, DE-3-2 was finally purified using RP-HPLC system on an Agilent 1260 HPLC system with a Zorbax C-18 column, and the eluted peptides were gathered separately in accordance with chromatographic peaks. Among all chromatographic fractions, eight peptides with RT of 8.919 min (TJP1), 9.189 min (TJP2), 12.112 min (TJP3), 13.829 min (TJP4), 14.209 min (TJP5), 17.237 min (TJP6), 19.772 min (TJP7), and 20.436 min (TJP8) showed high radical scavenging activities. Therefore, TJP1-TJP8 were collected and lyophilized for amino acid sequence identification and activity evaluation.

### 2.3. Amino Acid Sequence Analysis and Mass Spectrometry of Peptides from Protein Hydrolysates of Hairtail Muscle

**Figure 7:**
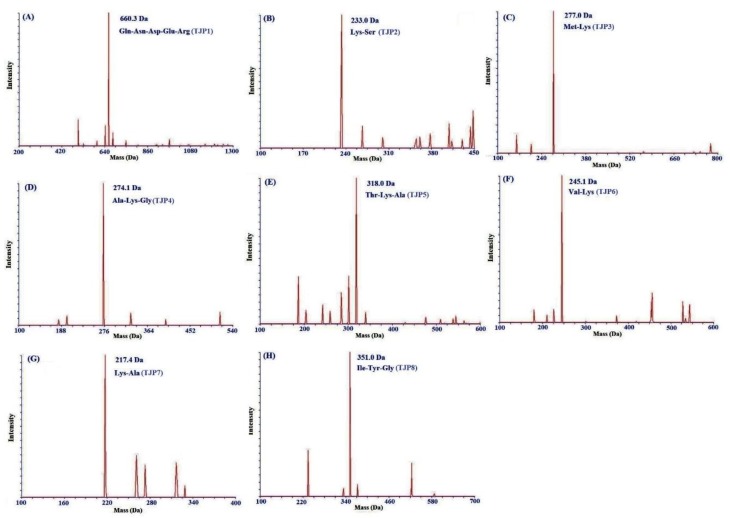
Mass spectra of TJP1 (A), TJP2 (B), TJP3 (C), TJP4 (D), TJP5 (E), TJP6 (F), TJP7 (G), and TJP8 (H) from protein hydrolysate of hairtail (*T. japonicas*) muscle.

For more detailed discussion on the structure-function relationship, the amino acid composition, sequences, and molecular mass of eight isolated peptides (TJP1-TJP8) were determined using protein sequencer and ESI-MS, and the results were shown in Figure 7 and Table 1. The amino acid sequences of eight isolated peptides (TJP1-TJP8) were identified as Gln-Asn-Asp-Glu-Arg (TJP1), Lys-Ser (TJP2), Lys-Ala (TJP3), Ala-Lys-Gly (TJP4), Thr-Lys-Ala (TJP5), Val-Lys (TJP6), Met-Lys (TJP7), and Ile-Tyr-Gly (TJP8) with molecular weights of 660.3, 233.0, 217.1, 274.1, 318.0, 245.1, 277.0, and 351.0 Da, respectively, which were agreed well with their theoretical masses of 660.6, 233.3, 217.3, 274.3, 318.4, 245.3, 277.4, and 351.4 Da.

### 2.4. Antioxidant Activity

**Figure 8:**
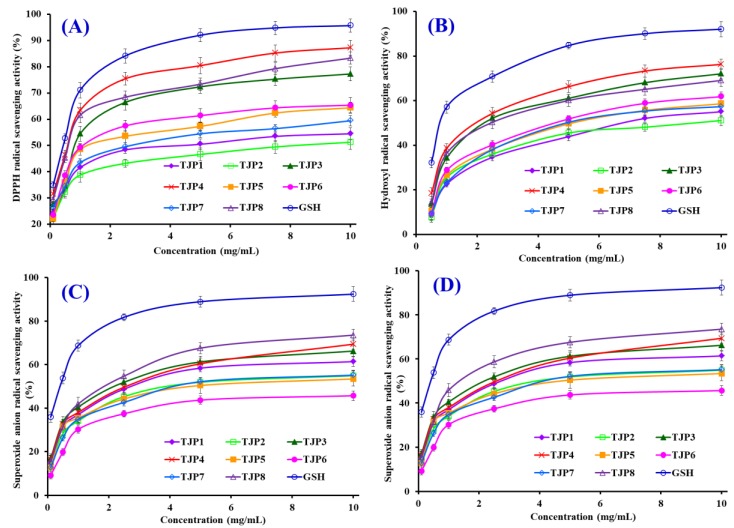
DPPH• (**A**); HO• (**B**); $O_{2}^{-}$• (**C**); and ABTS^+^• (**D**) scavenging activities of eight isolated peptides (TJP1 to TJP8) from protein hydrolysate of hairtail (*T. japonicas*) muscle. All data are presented as the mean ± SD of triplicate results.

**Figure 9:**
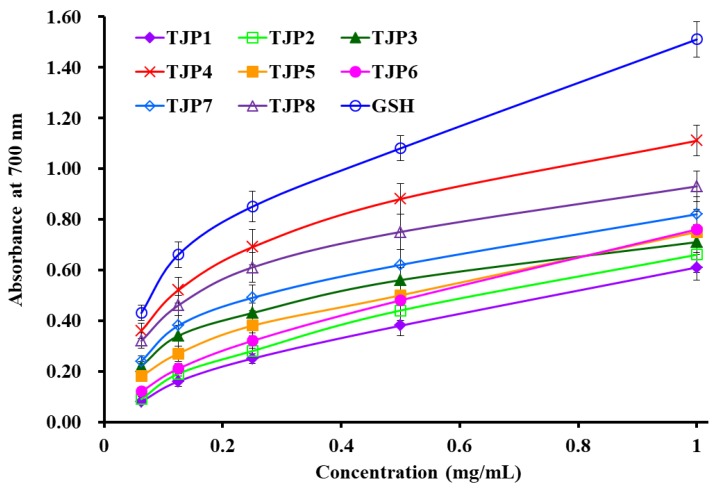
Reducing power of eight isolated peptides (TJP1 to TJP8) from protein hydrolysate of hairtail (*T. japonicas*) muscle. All data are presented as the mean ± SD of triplicate results.

**Figure 10:**
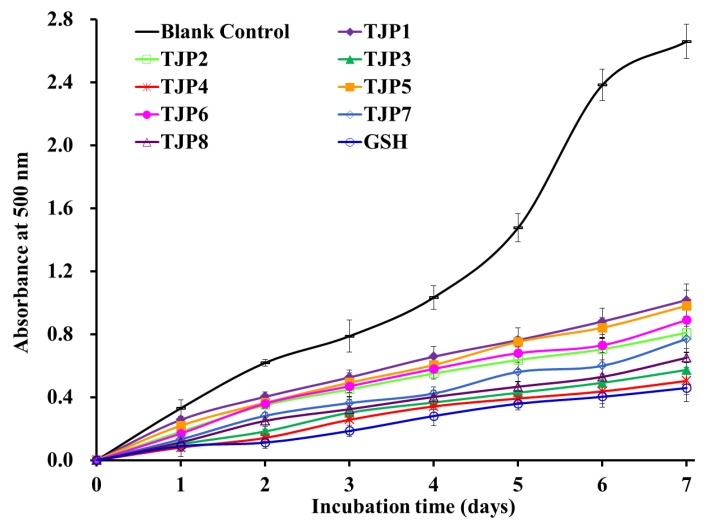
Lipid peroxidation inhibition assays of eight isolated peptides (TJP1 to TJP8) from protein hydrolysate of hairtail (*T. japonicas*) muscle. All data are presented as the mean ± SD of triplicate results.

To better evaluate the antioxidant activity of eight isolated peptides (TJP1 to TJP8) from protein hydrolysate of hairtail (*T. japonicas*) muscle, four kinds of radical (DPPH•, HO•, $O_{2}^{-}$•, and ABTS^+^•) scavenging assays, reducing power, and lipid peroxidation inhibition assay were tested, and the results were presented in Table 2 and Figure 8, Figure 9 and Figure 10.

#### 2.4.1. Radical Scavenging Activity

##### DPPH• Scavenging Activity

DPPH• shows maximal absorbance at 517 nm in its oxidized form, and the absorbance wears off with the free radical accepting an electron [3]. As shown in Figure 8A, eight isolated peptides (TJP1 to TJP8) showed strong DPPH• scavenging activities and there was also a positive correlation between the concentration and the radical-scavenging activity. The half elimination ratio (EC_50_) values of TJP3, TJP4, and TJP8 were 0.902, 0.626, and 0.663 mg/mL, respectively, and TJP4 exhibited the highest DPPH• scavenging ability among eight isolated peptides, but its activity was still lower than that of the positive control of glutathione (GSH) at the same concentration. The EC_50_ value of TJP4 was lower than those of most antioxidant peptides from protein hydrolysates of loach (PSYV: 17.0 mg/mL) [39], miiuy croaker (GIEWA: 0.78 mg/mL) [6], scalloped hammerhead cartilage (GPE: 2.43 mg/mL; GARGPQ: 2.66 mg/mL; GFTGPPGFNG: 1.99 mg/mL) [12], blue mussel (YPPAK: 2.62 mg/mL) [26], bluefin leatherjacket (GPP: 1.927 mg/mL; WEGPK: 4.438 mg/mL; GVPLT: 4.541 mg/mL) [8,14], skate cartilages (FIMGPY: 2.60 mg/mL; GPAGDY: 3.48 mg/mL; IVAGPQ: 3.93 mg/mL) [24], grass carp skin (GFGPL: 2.249 mg/mL; VGGRP: 2.937 mg/mL) [40], and salmon pectoral fin (TTANIEDRR: 2.503 mg/mL) [41]. However, the EC_50_ value of TJP4 was higher than those of peptides from protein hydrolysates of Chinese leek (GSQ: 0.61 mg/mL) [42], miiuy croaker (FPYLRH: 0.51 mg/mL) [6], grass carp skin (HFGBPFH: 0.20 mg/mL) [40], and skate muscle (APPTAYAQS: 0.614 mg/mL; NWDMEKIWD 0.289 mg/mL) [10]. Therefore, these data indicated that eight isolated peptides (TJP1 to TJP8), especially TJP3, TJP4, and TJP8 had the strong capacity to contribute an electron or hydrogen radical for suppressing the DPPH• reaction.

##### HO• Scavenging Activity

HO• is a highly reactive radical to the organism and only can be eliminated by endogenous and dietary antioxidants. Therefore, it is the ideal assay for searching the radical scavenging agent of organism. The abilities of eight isolated peptides (TJP1 to TJP8) were investigated, and the dose-related effects were observed at different peptide concentrations ranging from 0 to 10.0 mg/mL (Figure 8B). EC_50_ values of TJP3, TJP4, and TJP8 were 1.740, 2.378, and 2.498 mg/mL, respectively, and TJP3 exhibited the highest HO• scavenging ability among all isolated peptides at the same concentration. EC_50_ value of TJP3 was lower than those of peptides from protein hydrolysates of bluefin leatherjacket heads (WEGPK: 5.567 mg/mL; GPP: 2.385 mg/mL; GVPLT: 4.149 mg/mL) [8], weatherfish loach (PSYV: 2.64 mg/mL) [39], skate cartilages (FIMGPY: 3.04 mg/mL; GPAGDY: 3.92 mg/mL; IVAGPQ: 5.03 mg/mL) [24], and grass carp skin (PYSFK: 2.283mg/mL; VGGRP: 2.055 mg/mL) [40]. However, the EC_50_ value of TJP3 was higher than those of antioxidant peptides from protein hydrolysates of miiuy croaker (FPYLRH: 0.68 mg/mL; GIEWA: 0.71 mg/mL) [6], spotless smoothhound cartilage (GAERP: 0.25 mg/mL; GEREANVM: 0.34 mg/mL; AEVG: 0.06 mg/mL) [3], blue mussel (YPPAK: 0.228 mg/mL) [26], giant squid (NGLEGLK: 0.313 mg/mL; NADFGLNGLEGLA: 0.612 mg/mL) [43], conger eel (LGLNGDDVN: 0.687 mg/mL) [44], and skate muscle (APPTAYAQS: 0.390 mg/mL; NWDMEKIWD 0.176 mg/mL) [10]. TJP3, TJP4, and TJP8 showed strong HO• scavenging ability, which indicated that it could serve as a HO• scavenger for decreasing or eliminating the damage caused by HO• in food industries and biological systems. 

##### $O_{2}^{-}$• Scavenging Assay

$O_{2}^{-}$• is the most common free radical generated in vivo, and can promote oxidative reaction to generate peroxy and hydroxyl radicals. Superoxide dismutase protects the cell from the deleterious effects of superoxides in living organisms. Therefore, it is important to search safe and efficient antioxidants for scavenging $O_{2}^{-}$•. Figure 8C indicated the $O_{2}^{-}$• scavenging ratios of eight isolated peptides (TJP1 to TJP8) drastically increased with increasing concentration ranging from 0.1 to 10 mg/mL, but their activities were still lower than that of glutathione (GSH) at the same concentration. EC_50_ values of TJP3, TJP4, and TJP8 were 2.082, 2.538, and 1.355 mg/mL, respectively. Therefore, TJP8 played a significant role in $O_{2}^{-}$• scavenging. EC_50_ value of TJP8 was lower than those of peptides from protein hydrolysates of miiuy croaker swim bladders (FYKWP:1.92 mg/mL; FTGMD:3.04 mg/mL; YLPYA:3.61 mg/mL; GFYAA:3.03 mg/mL; FSGLR:3.35 mg/mL; VPDDD:4.11 mg/mL) [6], bluefin leatherjacket heads (WEGPK: 3.223 mg/mL; GPP: 4.668 mg/mL; GVPLT: 2.8819 mg/mL) [8], and skate cartilage (FIMGPY: 1.61 mg/mL; GPAGDY: 1.66 mg/mL; IVAGPQ: 1.82 mg/mL) [24]. However, EC_50_ value of TJP8 was higher than those of protein hydrolysates of croceine croaker muscle (YLMR: 0.450 mg/mL; VLYEE: 0.693 mg/mL; MILMR: 0.993 mg/mL) [32], skate muscle (APPTAYAQS: 0.215 mg/mL; NWDMEKIWD 0.132 mg/mL) [10], *Mytilus coruscus* (SLPIGLMIAM: 0.3168 mg/mL) [45], miiuy croaker swim bladders (GFEPY: 0.87 mg/mL; FPPYERRQ: 0.68 mg/mL; FPYLRH: 0.34 mg/mL; GIEWA: 0.30 mg/mL) [6], round scad (HDHPVC: 0.265 mg/mL; HEKVC: 0.235 mg/mL) [46], monkfish muscle (EWPAQ: 0.624 mg/mL; FLHRP: 0.101 mg/mL; LMGQW: 0.042 mg/mL) [47], and croceine croaker scales (GFRGTIGLVG: 0.463 0.151 mg/mL; GPAGPAG: 0.099 mg/mL; GFPSG: 0.151 mg/mL) [48]. $O_{2}^{-}$• is catalyzed into hydrogen peroxide and oxygen by superoxide dismutases (SOD) in organism. Therefore, TJP3, TJP4, and TJP8 can be applied to eliminate $O_{2}^{-}$• damage together with SOD in biological systems.

##### ABTS^+^• Scavenging Assay

The ABTS^+^• is reactive towards most antioxidants and the blue ABTS^+^• with an absorption maximum of 734 nm is converted back to its colorless neutral form during this reaction [3]. Therefore, ABTS^+^• scavenging assay is one of the most widely assay used to screen anti-radical peptides. As shown in Figure 8D, eight isolated peptides (TJP1 to TJP8) showed strong ABTS^+^• scavenging activities in a dose-effect manner with EC_50_ values of 1.925, 2.496, 1.652, 0.831, 3.527, 2.835, 8.752, and 0.586 mg/mL, respectively. TJP4 and TJP8 showed the strongest ABTS^+^• scavenging activity among eight isolated peptides, but still weaker than glutathione (GSH) did at the same concentration. The EC_50_ values of TJP4 and TJP8 were significantly lower than those of peptides from protein hydrolysates of salmon (FLNEFLHV: 1.548 mg/mL) [41], skate cartilages (FIMGPY: 1.04 mg/mL; IVAGPQ: 1.29 mg/mL) [24], bluefin leatherjacket heads (WEGPK: 5.407 mg/mL; GPP: 2.472 mg/mL; GVPLT: 3.124 mg/mL) [8], and corn gluten meal (FLPF: 1.497 mg/mL; LPF: 1.013 mg/mL; LLPF: 1.031 mg/mL) [49]. However, The EC_50_ values of TJP4 and TJP8 were significantly higher than those of peptides from protein hydrolysates of skate cartilages (GPAGDY: 0.77 mg/mL) [24], grass carp skin (GFGPL: 0.328 mg/mL; VGGRP: 0.465 mg/mL) [40], scalloped hammerhead cartilage (GPE: 0.24 mg/mL; GARGPQ: 0.18 mg/mL; GFTGPPGFNG: 0.29 mg/mL) [12], and *Sphyrna lewini* muscle (WDR:0.34 mg/mL; PYFNK: 0.12 mg/mL) [33]. These results indicated that eight isolated peptides (TJP1 to TJP8), especially TJP4 and TJP8, have the strong ability to convert ABTS^+^• to its colorless neutral form and hold back the radical chain reaction.

#### 2.4.2. Reducing Power

The reducing power is an important indicator for evaluating the activities of antioxidant peptides [30]. As shown in Figure 9, eight isolated peptides (TJP1 to TJP8) exhibited dose-dependent reducing power at the concentrations ranged from 0.5 mg/mL to 10 mg/mL, and TJP4 showed the higher capacity to reduce ferric ions (Fe^3+^) to ferrous ions (Fe^2+^) than other seven antioxidant peptides. However, the reducing power of eight isolated peptides (TJP1 to TJP8) was lower that of the positive control of GPS. 

#### 2.4.3. Lipid Peroxidation Inhibition Assay

In the experiment, DPPH•, HO•, $O_{2}^{-}$•, and ABTS^+^• scavenging assays have been used to assess the antioxidant activities of eight isolated peptides (TJP1 to TJP8), but oxidative process in biological systems or food products is complicated and embroiled in different kinds of reactions for propagation of lipid radicals and lipid hydroperoxides in the presence of oxygen [3,37]. The radical scavenging assays only measured an antioxidant property, which cannot reflect its role as an antioxidant to protect organism and/or food systems from lipid oxidation [3,32]. As a consequence, we investigated the abilities of eight isolated peptides (TJP1 to TJP8) to control lipid peroxidation in a linoleic acid model system, and the result was presented in Figure 10. The absorbance at 500 nm of sample solutions with eight isolated peptides (TJP1 to TJP8), respectively, was significantly lower than that of the negative control (without antioxidant), and TJP3, TJP4, and TJP8 revealed similar abilities on lipid oxidation inhibition to that of positive control of GSH. The presented results demonstrated that eight isolated peptides (TJP1 to TJP8) could effectively hold back lipid peroxidation in the tested system during seven days of incubation. Furthermore, eight isolated peptides (TJP1 to TJP8) were isolated from the food resource of hairtail (*T. japonicas*) muscl and considered safer than chemical antioxidants. Therefore, we can increase the using dose of isolated peptides (TJP1 to TJP8) to compensate the flaw that their antioxidant activity is lower than that of chemical antioxidants.

## 3. Discussion

At present, hundreds of antioxidant peptides have been isolated from different resources. However, there is still insufficient evidence to elucidate the structure-activity relationship of antioxidant peptides. Generally, molecular size, hydrophobicity, and amino acid composition and sequence are deemed to play key roles in their antioxidant capacities [7,50].

Bioactivities of antioxidant peptides are highly dependent on their molecular size because small antioxidants have the higher possibility to interact with free radicals to prevent the lipid peroxidation [3,33]. Furthermore, smaller peptides are more likely to pass through the blood-brain barrier to perform their physiological functions in the body and have the high possibility to develop into new drugs [30,51]. Therefore, shorter size peptides especially peptides with 2–10 amino acid residues are deemed to obtain stronger radical scavenging and lipid peroxidation inhibition activities than long-chain peptides [6,7]. In the study, eight isolated peptides (TJP1 to TJP8) from protein hydrolysate of hairtail muscle are dipeptides (TJP2, TJP3, TJP6, and TJP7), tripeptides (TJP4, TJP5, and TJP8), or pentapeptide (TJP1), which help them to contact the target more easily to exert their bioactive properties.

More importantly, amino acids play a critical role in the antioxidant of peptides. Sila and Bougatef reported that hydrophobic amino acids, such as Leu, Ile, Ala, Val, and Met, had high reactivity to hydrophobic PUFAs and exert their significant effects on radical scavenging in lipid-rich foods [7,33]. Zhao et al. presented that the Ile and Ala residues contribute to the lipid peroxidation inhibitory and radical-scavenging and activities of GIEWA [6]. Therefore, Ala in the sequence of TJP4 and Ile residues in the sequence of TJP8 should positively influence its antioxidant activity. Dávalos et al. [52] and Xing et al. [53] confirmed that Met residue showed the highest antioxidant activity among all the amino acids because its large hydrophobic group can help peptides to facilitate the contacts with hydrophobic radical species. Wu et al. further reported that Met residue in PMRGGGGYHY might work as a reactive site, where the peptide could scavenge oxidants through the formation of a sulfoxide structure after oxidation to stop free-radical chain reactions [54]. Moreover, aromatic residues of aromatic amino acids—including Phe, Trp, and Ty—can keep radical stable during the scavenging process through contributing protons to electron deficient radicals [38]. Tyr residues could turn free radicals into more stable phenoxy radicals to stop the peroxidizing chain reaction [55]. Therefore, Met residues in the sequences of TJP3 and Tyr residues in the sequences TJP8 should be the important contributors for the antioxidant activity of TJP3 and TJP8. 

Polar amino acids is reported to play a critical role in HO• scavenging and metal ion chelating activities because of their carboxyl and amino groups in the side chains [56,57]. Zhu et al. reported that peptides consisted of Lys, Glu, and Asp were identified to have strong abilities to chelate metal ions as well as scavenge HO• [57]. Ren et al. have reported that basic peptides had greater capacity to scavenge HO• than acidic or neutral peptides [58]. Hu et al. reported that the presence of basic amino acid of Lys was one of the main reasons for the antioxidant activity of NWDMEKIWD [10]. Gly residue is found to contribute significantly to antioxidant activity since the single hydrogen atom in its side chain can provide a high flexibility to the peptide backbone, serving as proton-donors and neutralizing active free radical species [59,60]. Therefore, polar amino acids including Lys and Gly residues could play a critical role in the radical scavenging activities of TJP3, TJP4, and TJP8.

## 4. Experimental Section

### 4.1. Materials

Hairtail (*T. japonicas*) muscle was purchased from Fengmao Market in Zhoushan city of China. Bovine serum albumin (BSA), DEAE-52 cellulose and Sephadex G-15 were purchased from Shanghai Source Poly Biological Technology Co., Ltd. (Shanghai, China). Acetonitrile (ACN) of LC grade and trifluoroacetic acid (TFA) were purchased from Thermo Fisher Scientific Co., Ltd. (Shanghai, China). Phosphate buffered saline (PBS, pH 7.2), DPPH, and ABTS were purchased from Sigma Chemicals Co. (USA). QNDER(TJP1), KS(TJP2), KA(TJP3), AKG(TJP4), TKA(TJP5), VK(TJP6), MK(TJP7), and IYG(TJP8) with purity higher than 98% were synthesized in China Peptides Co. (Suzhou, China). All other reagents were analytical grade and purchased from Sinopharm Chemical Reagent Co., Ltd. (Shanghai, China).

### 4.2. Preparation of Protein Hydrolysate from Hairtail (T. japonicas) Muscle

The hairtail (*T. japonicas*) muscle was homogenized and blended with isopropanol at a ratio of 1:4 (w/v) and stand at 30 ± 2 °C for 1 h. The supernatant was drained and the residue was defatted using isopropanol at a ratio of 1:4 (w/v) at 75 ± 2 °C for 90 min. Finally, the supernatant was then removed and the solid precipitate was air-dried at 35 ± 2 °C.

The resulted precipitate was dispersed in distilled water (DW) at a ratio of 1:10 (w/v), and hydrolyzed separately using trypsin at pH 7.8, 37.5 °C, alcalase at pH 8.5, 50 °C, neutrase at pH 7.0, 50 °C, papain at pH 7.0, 50 °C and pepsin at pH 2.0, 37.5 °C with total enzyme dose 2% (w/w, 2 g enzyme/100 g defatted precipitate powder). In 4 h, the protein hydrolysates were heated to 95 °C for 10 min and centrifuged at 12,000 *g* for 15 min, and the five supernatants were lyophilized. Protein hydrolysates prepared separately using alcalase and papain exhibited the highest HO• scavenging activity among five protein hydrolysates. Therefore, defatted precipitate of hairtail muscle was scattered in DW at a ratio of 1:5 (w/v), and hydrolyzed using papain and alcalase for 5 and 6 h successively on above hydrolysis conditions. The resulted hydrolysate was treated in the same manner as the above method and referred to as HTP. 

The concentrations of protein hydrolysate and its fractions were expressed as mg protein/mL and measured by the dye binding method of Bradford (1976) with BSA as the standard protein.

### 4.3. Isolation of Peptides from HTP

#### 4.3.1. Fractionation of HTP by Ultrafiltration

HTP was fractionated using ultrafiltration (8400, Millipore, Hangzhou, China) with 1, 3, and 5 kDa MWCO membranes (Millipore, Hangzhou, China), and four fractions termed HTP-I (MW < 1 kDa), HTP-II (MW 1–3 kDa), HTP-III (MW 3–5 kDa), and HTP-IV (MW > 5 kDa) were collected and lyophilized.

#### 4.3.2. Anion-Exchange Chromatography

HTP-I solution (5 mL, 40.0 mg/mL) was injected into a DEAE-52 cellulose column (1.6 × 80 cm) pre-equilibrated with DW, and stepwise eluted with 150 ml DW, 0.1 M NaCl, 0.5 M NaCl, and 1.0 M NaCl solution at a flow rate of 1.0 mL/min, respectively. Each eluate (5 mL) was monitored at 280 nm. Finally, five fractions (DE-1 to DE-5) were pooled and lyophilized on the chromatographic peaks.

#### 4.3.3. Gel Filtration Chromatography

DE-3 solution (5 mL, 10.0 mg/mL) was separated on a Sephadex G-15 column (2.6 × 160 cm) eluted with DW at a flow rate of 0.6 mL/min. Each eluate (3 mL) was collected and monitored at 280 nm, and fraction of DE-3-2 with higher activity than others were collected and lyophilized.

#### 4.3.4. RP-HPLC

DE-3-2 was further purified on an Agilent 1260 HPLC system (Agilent Ltd., Santa Rosa, California, USA) with a Zorbax, SB C-18 column (4.6 × 250 mm). The sample was eluated with a linear gradient of acetonitrile (0–50% in 0–40 min) in 0.1% TFA at a flow rate of 0.8 mL/min. Eight peptides (TJP1 to TJP8) were isolated on the absorbance at 280 nm and lyophilized.

### 4.4. Determination of Amino Acid Sequence and Molecular Mass

The amino acid sequences and molecular masses of eight isolated peptides (TJP1 to TJP8) was measured on an Applied Biosystems 494 protein sequencer (Perkin Elmer/Applied Biosystems Inc., Foster City, CA, USA) and a Q-TOF mass spectrometer coupled with an electrospray ionization source (ESI), respectively. 

### 4.5. Antioxidant Activity

The DPPH•, HO•, $O_{2}^{-}$•, and ABTS^+^• scavenging activities of eight isolated peptides (TJP1 to TJP8) were measured on the previous method [52], and the EC_50_ was defined as the concentration where a sample caused a 50% decrease of the initial concentration of radical. The reducing power assay of eight isolated peptides (TJP1 to TJP8) was determined by the description of a literature report [30]. The lipid peroxidation inhibition assay of eight isolated peptides (TJP1 to TJP8) were determined in a linoleic acid model system on the method of Wang et al. [33]. 

### 4.6. Statistical Analysis

The data are reported as the mean ± standard deviation (SD) with three determinations. A one-way analysis of variance (ANOVA) test for differences between means of each group was applied to analyzed data using SPSS 19.0 (Statistical Program for Social Sciences, SPSS Corporation, Chicago, IL, USA). A *p*-value of less than 0.05 was considered statistically significant.

## 5. Conclusions

In the experiment, eight isolated peptides (TJP1 to TJP8) from protein hydrolysate of hairtail (*T. japonicas*) muscle prepared with alcalase + papain were isolated and identified as QNDER (TJP1), KS (TJP2), KA (TJP3), AKG (TJP4), TKA (TJP5), VK (TJP6), MK (TJP7), and IYG (TJP8), respectively, which exhibited high antioxidant activities through radical scavenging, reducing power, and lipid peroxidation inhibition assays. On the present results, the peptide fractions and isolated peptides (TJP1 to TJP8) from protein hydrolysate of hairtail (*T. japonicas*) muscle may be applied as an ingredient in new functional foods, and detailed studies will be done to illustrate the relationship between the activities and structures of eight isolated peptides. In addition, animal feeding experiments on isolated peptides (TJP1 to TJP8) will be conducted to evaluate their in vivo antioxidant effects.

## Figures and Tables

**Table 1:** Retention time, amino acid sequences, and molecular mass of eight isolated peptides (TJP1 to TJP8) from protein hydrolysate of hairtail (*T. japonicas*) muscle.

	Retention time (min)	Amino acid sequence	Theoretical mass/observed mass (Da)
TJP1	8.919	Gln-Asn-Asp-Glu-Arg	660.3/660.6
TJP2	9.189	Lys-Ser	233.0/233.3
TJP3	12.112	Lys-Ala	217.1/217.3
TJP4	13.829	Ala-Lys-Gly	274.1/274.3
TJP5	14.209	Thr-Lys-Ala	318.0/318.4
TJP6	17.237	Val-Lys	245.1/245.3
TJP7	19.772	Met-Lys	277.0/277.4
TJP8	20.436	Ile-Tyr-Gly	351.0/351.4

**Table 2:** Radical scavenging activity of eight isolated peptides (TJP1 to TJP8) from protein hydrolysate of hairtail (*T*. *japonicas*) muscle.

		EC_50_ (mg/mL)		
	DPPH•	HO•	$O_{2}^{-}$•	ABTS^+^•
TJP1	4.95	6.865	2.753	1.925
TJP2	7.68	5.634	4.296	2.496
TJP3	0.902	1.740	2.082	1.652
TJP4	0.626	2.378	2.538	0.831
TJP5	1.425	5.261	4.911	3.527
TJP6	1.262	3.845	>10.000	2.835
TJP7	3.150	4.993	4.427	8.752
TJP8	0.663	2.498	1.835	0.586
Glutathione (GSH)	0.251	0.758	0.456	0.078
